# A clinicopathologic study on calcifying epithelial odontogenic tumor: with special reference to Langerhans cell variant

**DOI:** 10.1186/1746-1596-9-37

**Published:** 2014-02-20

**Authors:** Yan Chen, Ting-Ting Wang, Yan Gao, Tie-Jun Li

**Affiliations:** 1 Department of Oral Pathology, Peking University School and Hospital of Stomatology, Beijing, China; 2 Department of Oral Medicine, Hebei United University, School and Hospital of Stomatology, Tangshan, Hebei, China; 3 Central Laboratory, Peking University School and Hospital of Stomatology, Beijing, China; 4 National Engineering Laboratory for Digital and Material Technology of Stomatology, Beijing, China

**Keywords:** Calcifying epithelial odontogenic tumour, Langerhans cell variant, Jaws, Histogenesis, Behaviour

## Abstract

**Background:**

Calcifying epithelial odontogenic tumour (CEOT) is a rare benign odontogenic tumour, and its Langerhans cell variant is even rarer. Due to the limited number of recorded cases, the biological behaviour and histogenesis of the Langerhans cell variant of CEOT are not yet fully understood. Thus, the correlation between conventional CEOT and the Langerhans cell variant remains to be clarified.

**Material (cases):**

Eight cases of CEOT including 2 cases of Langerhans cell variant were clinicopathologically studied and the English language literature was reviewed. Langerhans cells were detected in 2 cases of conventional CEOT and in 2 cases of Langerhans cell variant by immunohistochemistry.

**Results and findings:**

In the 6 cases of conventional CEOT, 5 tumours involved the premolar and molar region and the anterior portion of the mandible was affected in 1 case. Four patients were followed for 2–7 years and did not show any sign of recurrence. A review of the English language literature revealed 5 cases; combined with the present 2 new cases, a total of 7 cases of Langerhans cell variant of CEOT were collected. The patients were all Asian. Six tumours occurred in the maxilla and 1 in mandible; all mainly involved the anterior region of the jaws. Five patients were followed for 2-10 years and did not show any evidence of recurrence. Langerhans cells can be seen in both the conventional and the Langerhans cell variant of CEOT; however, increased numbers of Langerhans cells are seen in the latter.

**Conclusions:**

Although the Langerhans cell variant of CEOT is a rare entity and behaves similarly to the conventional type, it could show unique clinical and histologic features that may pose problems for differential diagnosis.

**Virtual slides:**

http://www.diagnosticpathology.diagnomx.eu/vs/1979090740113894

## Introduction

Calcifying epithelial odontogenic tumour (CEOT), also known as Pindborg tumour, is a rare benign odontogenic tumour that is characterized by the presence of amyloid material that may become calcified [1,2]. It is uncommon, accounting for only approximately 1% of all odontogenic tumours. Histologically, the classic form shows sheets and strands of variable size, polyhedral epithelial cells with well-defined borders, and distinct intercellular bridges. Nuclear pleomorphism and hyperchromatism are common. Round eosinophilic homogeneous deposits or amyloid substances are frequently found and often calcified. A pattern of concentric calcification called Liesegang rings is common, but it is not always present [3].

Interestingly, despite the small number of documented cases of CEOT, several variants of this tumour have been reported, including the presence of clear cells [3-5], Langerhans cells [6,7], myoepithelial cells [3], bone, and cementum-like components [8]. There have also been reports of CEOT combined with adenomatoid odontogenic tumours (AOTs) [3] or ameloblastoma [9].

Asano et al. [6] and Takata et al. [7] described the Langerhans cell variant of the intraosseous CEOT in 2 Japanese patients. In both cases, the tumour chiefly consisted of scattered small islands of epithelial cells. In some nests, a few clear cells tested positive for S-100 protein, lysozome, MT 1, LN-3, and OKT 6 antibodies but not for keratin antibody. Almost no calcification of homogenous eosinophilic materials was observed. Ultrastructurally, the S-100 positive cells were identified as Langerhans cells based on the finding of rod- and racket-shaped Birbeck’s granules. Three more Asian cases were also reported later [10,11].

Due to the limited cases, the biological behaviour and histogenesis of the Langerhans cell variant of CEOT are not yet fully understood, and the relationship between normal CEOT and the Langerhans cell variant remains to be clarified. Here, we report 8 cases of CEOT, including 2 cases of the Langerhans cell variant, from our own file and review the literature with special reference to the Langerhans cell variant.

## Material and methods

Cases diagnosed as CEOT or cysts with CEOT-like hyperplasia from January 1985 to December 2010 were retrieved from the files of the Department of Oral Pathology, Peking University School of Stomatology. The study protocol was approved by the Ethical Committee for Human Experiments of Peking University School of Stomatology (IRB00001052-11041). Standard hematoxylin and eosin-stained sections were reviewed and the lesions were reclassified according to the World Health Organization Classification of Odontogenic Tumours [12].

Eight cases of CEOT were confirmed: 6 cases had typical histopathological features similar to the description of CEOT, while the other 2 cases showed unusual histopathological features similar to the description of the Langerhans cell variant of CEOT in the literature. After confirmation by immunohistochemical staining, the 2 cases were classified as the Langerhans cell variant of CEOT. Clinical data including age, sex, anatomic site, duration, radiographic features, clinical impression, treatment, and available follow-up data were recorded. Cases of the Langerhans cell variant of CEOT reported in the English language literature were reviewed.

Two cases of the Langerhans cell variant were analysed by immunohistochemistry. Four-μm-thick tissue sections were deparaffinised and rehydrated. Endogenous peroxidase activity was blocked in 3% H_2_O_2_. For antibodies against human S-100, CD 1a, and langerin, antigen retrieval was achieved by treating the sections with a boiling solution of tris-ethylenediaminetetraacetic acid (pH 8.0) for 10 min in a microwave. Except for the anti-langerin antibody (Maixin; Fuzhou, Fujian, China), the primary antibodies were purchased from Zhongshan Biotechical Company (Beijing, China). The colour was developed in freshly made diaminobenzidine. The sections were washed briefly in running tap water and lightly stained with Mayer’s hematoxylin. Negative controls were prepared by omission of the relevant primary antibody.

To compare the distribution of Langerhans cells in the 2 cases of the Langerhans cell variant of CEOT, 2 cases of typical CEOTs (Table 1, cases 1 and 4) were also analysed by immunohistochemistry. Sections of a case of Langerhans cell histiocytosis were used as positive control. The numbers of langerin-positive cells and epithelial tumour cells from 5 randomly selected areas of each section under high power magnification (× 400) in each case were scored to obtain the ratio of Langerhans cells to epithelial tumour cells.

**Table 1:** The clinical information of conventional CEOT

**Case**	**Age (year)/sex**^**a**^	**Duration (month)**	**Location**^**b**^	**Symptom**	**Oral examination**	**Association with impact teeth**	**Radiographic feature**	**Resorption of teeth root**	**Treatment and follow-up**^**c**^
1	22/M	10	Max, R6 to tubera	Swelling	Swelling, no loose teeth	Yes	Unilobular, radiolucent and radiopaque	No	Enucleation, 7 year after surgery, NER
2	20/M	12	Mand, L6-8	Swelling	Swelling, no loose teeth	No	Unilobular, radiolucent	No	Enucleation, 7 year after surgery, NER
3	39/M	2	Mand, L3-5	Swelling	Swelling, loose teeth	No	Multilobular, radiolucent	No, displace of 4,5	Enucleation, lost to follow-up
4	37/F	0	Mand, L6 to retramolar region	No symptom	Swelling, no loose teeth	No	Unilobular, radiolucent and radiopaque	No	Segmental resection, 4 year after surgery, NER
5	41/F	3	Mand, L4-6	Swelling	Swelling, no loose teeth	Yes	Unilocular, radiolucent	No	Curretage, 2 year after surgery, NER
6	12/F	1	Mand, L4-R5	Swelling	Swelling, no loose teeth	Yes	Unilocular, radiolucent and radiopaque	No	Currettage, no follow-up information

^a^M = male, F = female; ^b^max = maxilla, mand = mandible, R = right, L = left; ^c^NER = no evidence for recurrence.

## Results

### Conventional CEOT

#### Clinical findings

**Figure 1:**
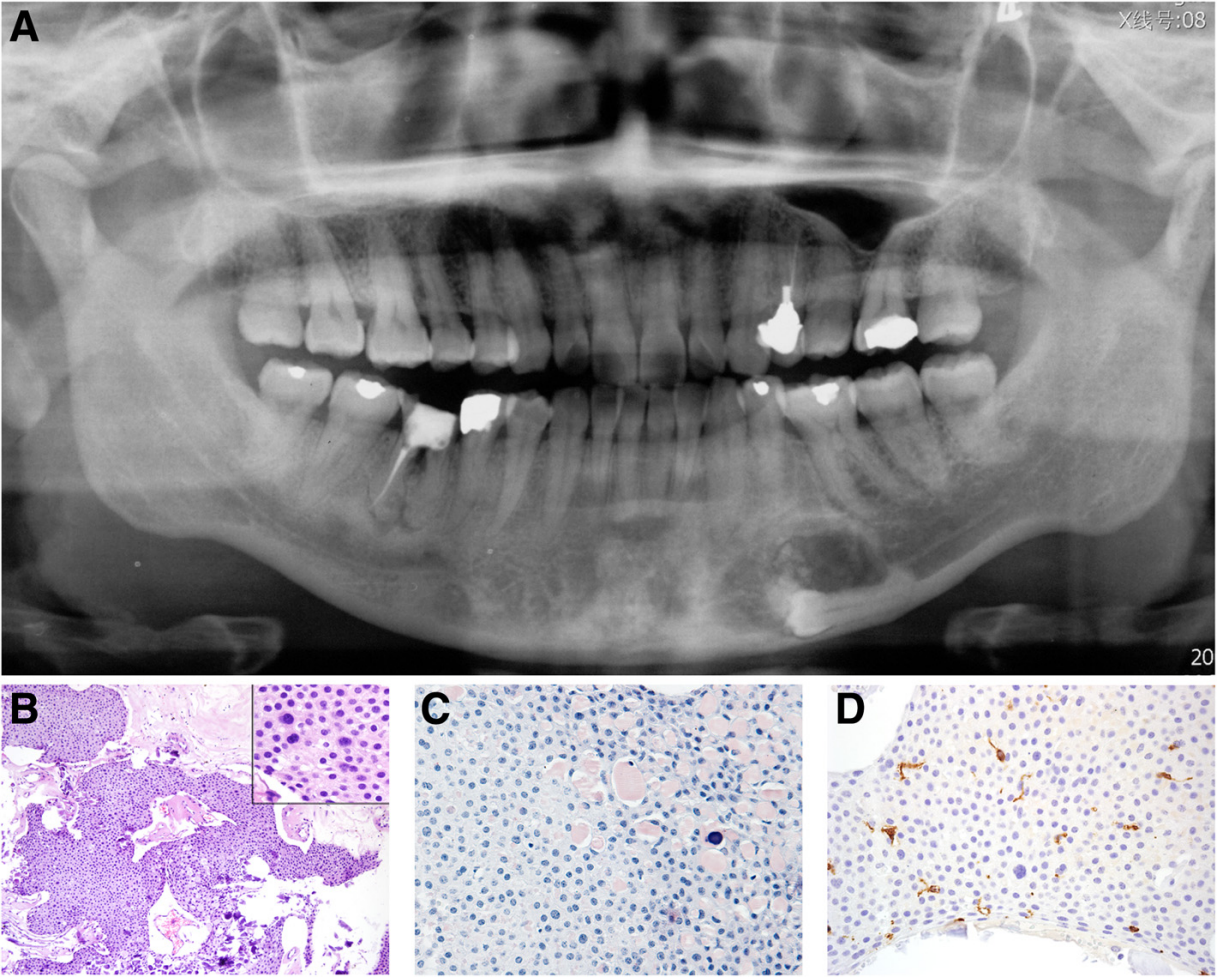
**Radiographic and histopathologic features of conventional CEOT. A**. A well-demarcated mixed radiolucent and radiopaque lesion is seen in the madible between the left canine and the first molar in close association with an unerupted premolar (case 5). **B**. The tumor was composed of masses of polyhedral epithelial cells with some eosinophilic amyloid-like surrounding material. Calcium salts were diffusely deposited. (HE, ×100). The tumor cells of conventional CEOT have distinct intercellular bridges and are irregular in shape with pleomorphic nuclei. (insert, HE, × 400). **C**. Eosinophilic amyloid-like surrounding material is positive for Congo red staining. Some amyloid-like material is undergoing calcification. (Congo red, ×200). **D**. Langerin positive cells scatter in the epithelial mass. (IHC, ×400).

The clinical features of 6 cases of classic CEOT are summarized in Table 1. The patient age at first presentation was 12–41 years (median age, 29.5 years). Three patients were male and 3 were female. Five tumours occurred in the mandible and 1 occurred in the maxilla. Five tumours involved the premolar and molar regions, while the anterior portion of the mandible was affected in 1 case. The first sign in 5 cases was jaw swelling, while the other case was incidentally discovered. In 5 patients, well-defined unilocular radiolucencies were noted, 3 of which had radiopaque areas and an unerupted tooth (Figure 1A). Five patients were initially treated by enucleation or curettage and 1 patient was managed with segmental resection. Four patients were followed for 2–7 years and none of them showed any sign of recurrence.

#### Pathological findings

On histological examination, the tumour was composed of strands, nests, and masses of polyhedral epithelial cells with eosinophilic cytoplasm and prominent nuclei. These cells had distinct intercellular bridges and were irregularly shaped with pleomorphic nuclei (Figure 1B). Between cells, there were round-shaped eosinophilic amyloid materials that stained positive for Congo red. The reddish homogeneous material appeared to undergo calcification and become concentric calcified corpuscles or calcified masses (Figure 1C). Langerin-positive cells were scattered in the epithelial mass of the tumour (Figure 1D). The ratio of langerin-positive cells to epithelial tumour cells was 1.7:100 and 0.8:100, respectively, in the 2 cases. S100 and CD1a staining showed the same pattern.

### Langerhans cell variant of CEOT

#### Case report

##### Case 1

A 40-year-old Chinese woman had a chief complaint of loosened left anterior teeth and pain that she first noted 4 years ago. The pain and loosened teeth had recently become obvious. Her past medical history was unremarkable with no evidence of systemic diseases. Intraoral examination revealed a depression over the left anterior hard palate. Loss of both labial and palatal alveolar bone was noted. The lateral incisor was loosened.

**Figure 2:**
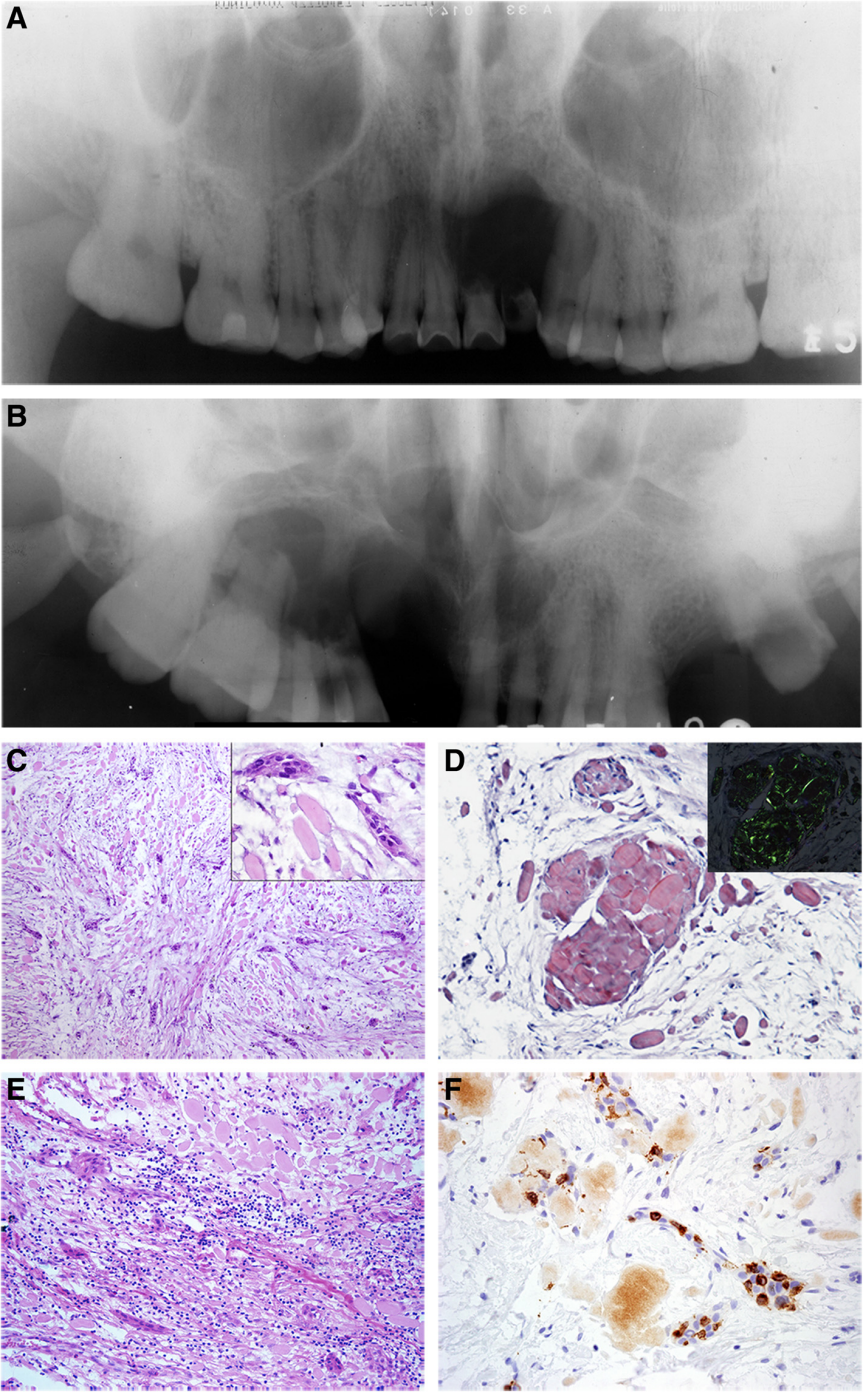
**Radiographic and histopathologic features of Langerhans cell variant of CEOT. A**. Radiographic examinations revealed unilocular, well defined, radiolucent areas from the right central incisor to the left first premolar. There were no radiopaque foci in the lesion. Resorption of the left central and lateral incisor was detected (case 1). **B**. A multilocular radiolucent lesion without a definite sclerotic border in part area from the left canine to the right second molar. No radiopaque foci were found and the roots of the right canine to the first molar were resorbed (case 2). **C**. The tumor was composed of small nests or strands of odontogenic epithelial cells and amorphous eosinophilic globules of amyloid-like materials in a loose fibrous connective tissue stroma. (HE, ×100) The small epithelial islands were composed of eosinophilic cytoplastic polyhedral tumor cells and a few clear cells. (insert, HE, ×400). **D**. The globular masses of homogeneous eosinophilic material is positive for Congo red staining (Congo red, ×200) and showed apple green birefringence when subjected to polarized light (insert, Congo red, ×200). **E**. Moderate chronic inflammatory cells in the fibrous connective tissue. (HE, ×200). **F**. Langerin-positive cells are seen in islands of epithelium, a higher Langerhans cells to epithelial tumor cells ratio can be seen (IHC, ×400).

Radiographic examination revealed a well-defined unilocular radiolucent area from the right central incisor to the first premolar (Figure 2A). There were no radiopaque foci within the lesion. Resorption of the left central and lateral incisor was detected. The clinical impression was a cyst. The lesion was curetted; during the surgery, however, the lesion was solid. The patient remained disease-free 5 years after the operation.

##### Case 2

A 58-year-old Chinese man was referred to our hospital for the evaluation of a swelling in the right maxilla that had been noted 3 months ago in a regular physical examination. There were no obvious symptoms or signs at the lesion site reported by the patient. The patient had no history of systemic disease but had suffered from allergenic rhinitis for >20 years. Intraoral examination revealed a loss of alveolar bone along with the missing right central and lateral incisors. The right canine was loose. There was a swelling of the plate from the right canine to the second molar area.

Radiographs revealed a multilocular radiolucent lesion without a definite sclerotic border from the left lateral incisor to the right second molar. The floor of the right maxillary sinus was not clear. No radiopaque foci were found in the lesion, while the roots of the right canine to the first molar were resorbed (Figure 2B). Ten years after a partial maxillectomy, no evidence of recurrence was found.

#### Pathological findings

These 2 tumours chiefly consisted of small nests and cords of epithelial cells and loose fibrous connective tissue stroma. The epithelial islands were composed of eosinophilic cytoplastic polyhedral tumour cells and a few clear cells (Figure 2C). The nuclei were relatively dense and contained a single nucleolus. Slight variation in cellular and nuclear size was seen. Intercellular bridges were occasionally seen between the tumour cells.

The globular masses of the homogeneous eosinophilic material tested positive for Congo red stain, were mostly located in the connective tissue, and showed apple green birefringence when subjected to polarized light (Figure 2D). There was moderate chronic inflammatory cell infiltration in the fibrous stroma (Figure 2E). No calcification was noted. Langerhans cells positive for langerin, S100, and CD1a were seen in the epithelial islands of the tumour (Figure 2F). The ratio of Langerhans cells to epithelial tumour cells was 82.7:100 and 42.1:100, respectively, in the 2 cases.

## Discussion

CEOT is an uncommon odontogenic tumour. Upon reviewing our own file of odontogenic tumours from 1985 to 2006, we found a total of 6 cases of odontogenic tumours among 1,309 cases (0.46%) [13]. The male:female ratio of patients with CEOT was 1:1. The tumours were primarily in the mandible (maxilla:mandible ratio, 1:2), and approximately 82% of the cases were located in the premolar and molar regions [3]. The clinical features of the present series were in general agreement with those of earlier reports, i.e. equal sex distribution and predilection for the mandible, especially the premolar and molar regions.

When first described [1], CEOT was considered a locally invasive tumour, and some subsequent publications supported this concept. This view was based upon evidence suggestive of bone marrow involvement from radiographs and histological sections [2,14]. However, Vap et al [14] believed that the tumour is an expansile lesion that does not extend into the intertrabecular spaces as does the infiltrative solid ameloblastoma. Franklin and Pindborg [15] reported a recurrence rate of 14% that was primarily attributed to inadequate treatment. A few cases of malignant transformation have been reported [16,17]. It is now accepted that CEOT is a less aggressive tumour than ameloblastoma that responds well to conservative surgery. Four cases of CEOT with follow-up data in the present series showed no signs of recurrence following surgery, which is in keeping with the notion.

As the first few reported cases of CEOT were all associated with an unerupted tooth, Pindborg [2] initially believed that CEOT develops from the reduced enamel epithelium of an unerupted tooth. However, as reports of cases without an associated unerupted tooth and cases of a peripheral variant started to accumulate, it became evident that sources other than reduced enamel epithelium should also be considered regarding CEOT histogenesis.

In the present series, 3 of 6 cases were associated with an impacted tooth. This is consistent with the literature that approximately half of CEOT cases are associated with an unerupted tooth. A few reports of a dentigerous cyst or dental follicle showed focal CEOT-like hyperplasia [18,19], which suggests that CEOT associated with the crowns of impacted teeth may indeed originate from the reduced enamel epithelium.

Our review of the English literature revealed 5 cases, together with our 2 new cases, for a total of 7 cases of the Langerhans cell variant of CEOT (Table 2). All of the patients were Asian. The patient age range at first presentation was 38–58 years (median, 44 years). Three patients were male and 4 were female. Six tumours occurred in the maxilla, while 1 occurred in the mandible. Five of the 6 maxillary tumours involved the anterior region. This feature is in contrast to the findings of conventional CEOT. Jaw swelling was noted in 4 cases. Two patients complained of loosened teeth and 1 patient showed no obvious symptoms. During an intraoral examination, bony depression of the tumour area was noted in 3 cases, a unique feature not seen in conventional CEOT.

**Table 2:** The clinical information of reported cases of Langerhans cell variant of CEOT

**Author (year)**	**Age (year)/sex**^**a**^	**Duration (month)**^**b**^	**Location**^**c**^	**Symptom**	**Oral examination**	**Association with impact teeth**	**Radiographic feature**	**Resorption of teeth root**	**Treatment and follow-up**^**d**^
Present series	58/M	3	Max, R molar to L3	Swelling	Swelling, loose teeth	No	Mutilobular radiolucent	R3-6	Partial resection of the maxilla, 10 years NER
Present series	40/F	48	Max, R incisor to L premolar	Loose teeth, pain	Depression over the anterior maxilla, loss of alveolar bone	No	Unilobular radiolucent	L1-2	Currettage, 5 years NER
Asano M (1990)	44/F	Several years	Max, L1-R6	Swelling	Swelling	No	Unilobular, radiolucent	R1-3	Partial resection of the maxilla, no follow-up information
Takata T (1993)	58/M	6	Max, L3-5	Loose teeth, loss of palatal and buccal alveolar bone	No swelling, loss of bone, loose teeth	No	Unilobular, radiolucent	L3-5	Enucleated,10 years NER
Li Wang (2006)	38/M	N.S.	Mand, R4 to ramus	Swelling, pain	N.S.	N.s	Well-difined radiolucencent	N.S.	Partial resection of the mandible, 2.5 year NER
	39/F	24	Max, L premolar, gingiva	Swelling in gingiva	Swelling in gingiva	N.s	N.S.	N.S.	Resected, 2 year NER
Wang YP (2007)	52/F	0	Max, R anterior region	No symptom	Depression over the anterior maxilla	No	Unilobular, radiolucent	No	Partial maxillectomy 23-16, No follow-up information

^a^M = male, F = female; ^b^N.S. = not stated; ^c^max = maxilla, mand = mandible, R = right, L = left; ^d^NER = no evidence for recurrence.

Of the 6 cases with radiographic records, well-defined unilocular radiolucency was noted in 5 cases and multilocular radiolucency was noted in one. The radiopaque area was not evident in any cases. Root resorption was detected in 4 cases. Three patients were initially treated by enucleation or curettage and the other 4 cases underwent partial jaw resection. Five patients were followed for 2–10 years and no recurrence was recorded. Thus, the Langerhans cell variant of CEOT may show similar behaviours to those of conventional CEOT.

Histologically, the Langerhans cell variant of CEOT is unusual. Although Congo red-positive homogenous amyloid material could be seen, there was no calcification. The tumour islands were composed of small numbers of epithelial cells with clear cells suggestive of Langerhans cells. Langerin is the protein expressed in Langerhans cells that is localized in the Birbeck granules present within the cytoplasm. Langerin staining, accompanied by S100 and CD1a staining, confirmed the presence of Langerhans cells in the tumour.

With regard to histogenesis, none of the 7 cases summarized here were associated with an unerupted tooth; thus, the Langerhans cell variant of CEOT is less likely to arise from a reduced enamel epithelium. The tumour was mainly located within the jaw bone with apparent root resorption of the apical part of the involved tooth. Other tissue origins like the rests of Malassez should be considered.

Another interesting finding of the present study was that Langerhans cells were detected not only in the Langerhans cell variant of CEOT but also in the epithelial nests of the conventional type. Similar reports have been presented by other groups [20]. Langerhans cells are bone marrow-derived cells that migrate into the oral epithelium and serve as antigen-presenting cells. Because both oral and odontogenic epithelia originate from the same oral ectoderm, it is possible that Langerhans cells may also migrate into the epithelial nests of odontogenic tumours. Thus, Langerhans cells may not exclusively exist in the Langerhans cell variant of CEOT. However, the number of Langerhans cell increased dramatically in the Langerhans cell variant of CEOT. The increased number of Langerhans cells may be associated with inflammation. Interestingly, 6 of the 7 cases of Langerhans cell variant here had a moderate number of lymphocytes and plasma cell infiltration. Asono [6] postulated that Langerhans cells may play an important role in antigen presentation or regression of CEOT.

Although Langerhans cells increases are due to immunogenic reactions and do not exist exclusively in this kind of CEOT, we continue to use the term “Langerhans cell variant of CEOT” to describe it since a limited number of cases have been reported. At this point, the use of another name may not be helpful for accumulating data about this kind of CEOT with special clinical and histopathological characteristics. A more suitable name may be adopted after more cases have been reported.

Differentiating conventional CEOT from the Langerhans cell variant is not difficult. The conventional type affects the premola and molar region as an asymptomatic slow-growing expansile mass and may feature an unerupted tooth. In contrast, lesions of the Langerhans cell variant have a predisposition for the anterior maxilla and a possibly overlying bone depression. Histopathologically, in the conventional type, the tumour consists of islands and sheets of polyhedral epithelial cells, eosinophilic homogenous amyloid substance, and calcified tissue. In the Langerhans cell variant, very small islands and cords of neoplastic cells with abundant amyloid substance and no calcification are characteristic features.

In conclusion, the Langerhans cell variant of CEOT is rare but consistent. To date, it has only been reported in Asian people, mainly in the anterior portion of the maxilla. This jaw tumour could present features of bone depression with loss of teeth and alveolar bone. The lesion comprises small epithelial islands and amorphous eosinophilic globules of amyloid materials with no apparent calcification. These features are somewhat different from those of conventional CEOT and should alert the pathologists to the differential diagnosis.

## Consent

Written informed consents were obtained from the two patients for publication and any accompanying images.

## Competing interest

The authors declare that they have no competing interests.

## Authors’ contributions

Study concepts and study design: TJL, Data acquisition: YC, TTW, Quality control of data and algorithms: TJL, Data analysis and interpretation: YC, TTW, Manuscript preparation: YC, TTW, Manuscript editing: TJL, Manuscript review: TJL. YC and TTW: co-first author, TJL: corresponding author. All authors read and approved the final manuscript.

## Authors’ information

Yan Chen and Ting-Ting Wang: co-first author.
